# Case Analysis of Water Gushing in a Deep Foundation Pit Caused by Local Defects

**DOI:** 10.3390/s24010245

**Published:** 2023-12-31

**Authors:** Shifan Qiao, Yiqi Liu, Chaobo Feng, Ziyong Cai, Daolong Chen, Fei Meng, Ping Xu

**Affiliations:** 1 Department of Civil Engineering, Central South University, Changsha 410075, China; qiaosf@csu.edu.cn (S.Q.); lyq5178678@126.com (Y.L.); fengchaobo@csu.edu.cn (C.F.); 164801006@csu.edu.cn (Z.C.); cdlongcsu@163.com (D.C.); 2 Sino-Hunan Overseas Engineering and Development Co., Ltd., Changsha 410200, China; 3 School of Civil Engineering, Xiangtan University, Xiangtan 411105, China; xup@xtu.edu.cn

**Keywords:** soft soil foundation pit, local defects, limit equilibrium, stability evaluation, safety factor against surge

## Abstract

Water gushing is a common engineering geological disaster in the process of foundation pit construction. Its successful judgment directly affects the safety of engineering construction. Taking the case of water gushing at the bottom of the foundation pitas as a research object, the mechanism and treatment of water gushing in foundation pits, the stability against water gushing, and its influencing factors are analyzed with a field investigation, field testing, and theoretical calculation. The calculation formula for the safety factor and critical thickness of the foundation pit against surges, considering the influence of multiple factors, is deduced. The influence of the height of the confined water level, the thickness of the water-resisting layer, the shear strength of the soil mass, the reinforcement depth of the soil mass in the pit, and the diameter of the bearing pile in the pit on the safety factor of the foundation pit surge are expounded. In addition, measures such as the reinforcement of the soil mass in the passive area in the pit, the increase in the thickness of the water-resisting layer, and the reduction in the confined water level are proposed to improve the anti-surge stability of the foundation pit. A new monitoring method is proposed for characterizing uplift deformation at the bottom of the pit without affecting normal construction. The research results show that: (1) the minimum safety factor against surges, considering multiple factors, is 1.455, and the critical thickness is 5.87 m, which is in line with specifications. (2) Measures such as reinforcing the soil in the passive zone of the pit, increasing the thickness of the water-insulating layer, and lowering the bearing pressure level are used to improve the stability of the pit against surges. (3) The pit, obtained by the pit bottom counter-pressure, pumping water out of the pit, and the timely construction of the pit bottom bedding to block the program, exhibits a measured maximum bottom plate pressure of 115.189 kPa, and the deformation corresponding to the method proposed in this paper is 1.406 mm, which is better disposed in the field. The research results provide a reference basis for the judgment of anti-surge stability of foundation pits and similar engineering applications.

## 1. Introduction

Water gushing in foundation pits refers to the phenomenon of piping, sand flow, soil flow, or a similar boiling water outflow, which is caused by the bottom slab of the foundation pit being pushed or broken by the action of pressurized water, as well as the infiltration and damage of the weak permeable layer. Water gushing is a common engineering geological disaster during the construction process of foundation pits. Once water gushing occurs, the foundation pit will face safety hazards, which are highly likely to cause engineering accidents [1,2]. Therefore, during the construction process of foundation pits, attention must be paid to groundwater issues.

At present, the traditional pressure balance method [3] is one of the main methods for calculating the anti-surge stability of foundation pits in Chinese regulations and is widely used in the design and construction of foundation pit engineering. However, in practical engineering, there are many cases where h_cr_ < h (h_cr_ is the critical thickness, and h is the thickness of the impermeable layer), and there is no sudden water gushing in the foundation pit, which is not consistent with the actual situation [4]. The main reason for this discrepancy is that some factors, such as soil shear strength, the excavation plane size effect, and the pile–soil interaction, which increase the anti-surge stability of foundation pits, are not considered in the traditional pressure balance method. Therefore, many scholars have modified the traditional pressure balance method to consider these factors. Liu et al. [5] and Ma et al. [6] considered the influence of soil shear strength and derived formulas for calculating the surge safety factor and the critical thickness of the impermeable layer. Hong [7] presented results from two centrifuge tests that were conducted to simulate multi-propped excavations in-flight (with and without piles) in soft clay destabilized by hydraulic pressure from an underlying sand aquifer. Hong [8] carried out a centrifuge test to validate constitutive models and model parameters, while Chen et al. [9] established a numerical model of PIP-braced excavation in Shanghai soft clay overlying a confined aquifer using the upper-bound finite element limit analysis (UBFELA) method. Sun et al. [10,11] analyzed the influence of the properties of the impermeable soil layer, the type and layout of pile foundation in the pit on anti-surge stability, and the failure modes of foundation pits using centrifugal model tests. They proposed three types of surge failure modes for a foundation pit with confined water and derived evaluation formulas for anti-surge stability in different modes. Li et al. [12] used a combination of model experiments and numerical simulation methods and found that changes in confined water levels had a significant impact on the anti-surge stability of the waterproof layer of the pit bottom. As the confined water level increased, the pit bottom was prone to uplift deformation and overall uplift failure. Cui [13] studied the distribution of superstatic pore pressure in double-layer land base soil under the condition of bearing water level decline and analyzed the influence of the nonlinearity of soil, the rate of change in bearing water, and the permeability coefficient and compression index on the pore pressure response. Qin [14] took the Changzhou subway deep foundation pit as an example and used PLAXIS to explore the influence of combined parameters. 

However, due to the complexity and comprehensiveness of the geological environment of foundation pit engineering, there are few existing studies that comprehensively consider the impact of soil shear strength, soil reinforcement in the passive zone, groundwater permeability, bearing piles, and pile–soil interaction on the anti-surge safety factor. Based on the case of water gushing at the Nansha Port Railway Tunnel Open Excavation Deep Foundation Pit Project in Guangzhou, this study first analyzes the causes and treatment evaluation of water gushing in foundation pits. Then, based on the limit equilibrium theory, a surge analysis model of the foundation pit is established. This model considers the combined effects of the self-weight and shear strength of the impermeable layer soil, passive soil reinforcement at the bottom of the pit, pressurized water-jacking force, groundwater permeability, and the pile–soil interaction. In order to improve the evaluation method for the stability of foundation pit surges, a calculation formula for the foundation pit anti-surge safety factor and critical anti-surge thickness is proposed. The method considers the influence of multiple factors, and the impact of changes in relevant parameters on the safety factor of the pit bottom surge resistance is analyzed. The research results can provide a reference for the evaluation of foundation pit surge resistance stability and its engineering applications.

## 2. Engineering Background

### 2.1. Project Overview and Geological Conditions

**Figure 1:**
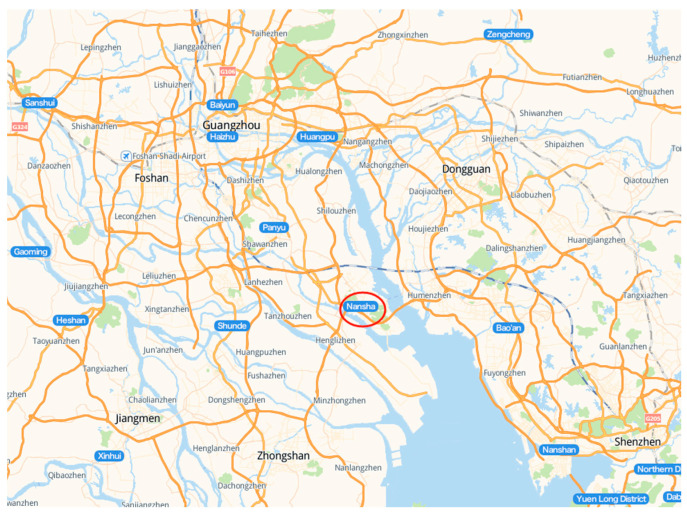
Field location map. The red circle marks the project site.

Located on Nansha District’s Longxue Island, the freshly inaugurated Guangzhou Nansha Port Railway Tunnel spans 6520 m from start to end (K0 500~K7 020), as illustrated in Figure 1. With its 4500 m subterranean segment crafted using open excavation, the tunnel features a longitudinally elongated excavation pit, varying from 0 to 14.9 m deep and averaging 7–9 m in width. The geological survey report outlines the properties of the distinct soil layers, including layer thickness (d), soil weight (γ), cohesion (c), internal friction angle (φ), and elastic modulus (E), as summarized in Table 1. Predominantly silt and muddy strata characterize the site, with an average layer thickness of 15.94 m. Geologically challenging, the entire tunnel lies on soft soil within the alluvial plain of the Pearl River Delta. This flat, expansive terrain houses shallow groundwater at an average buried depth of about 1.7 m. Confined water existing primarily in fine sand, coarse sand, and fine round gravel soils can also be found, with notable linkage to phreatic water. The excavation zone dewatering follows a segmented design, placing drainage wells every 15 m inside the pit and pressure-reducing wells 30–50 m apart outside the pit. These measures ensure groundwater is below the safe water level during pit excavation while monitoring sand flow and water gush. Classified as Level 1, the foundation pit demands utmost attention to safety.

### 2.2. Design of Bracing System

**Figure 2:**
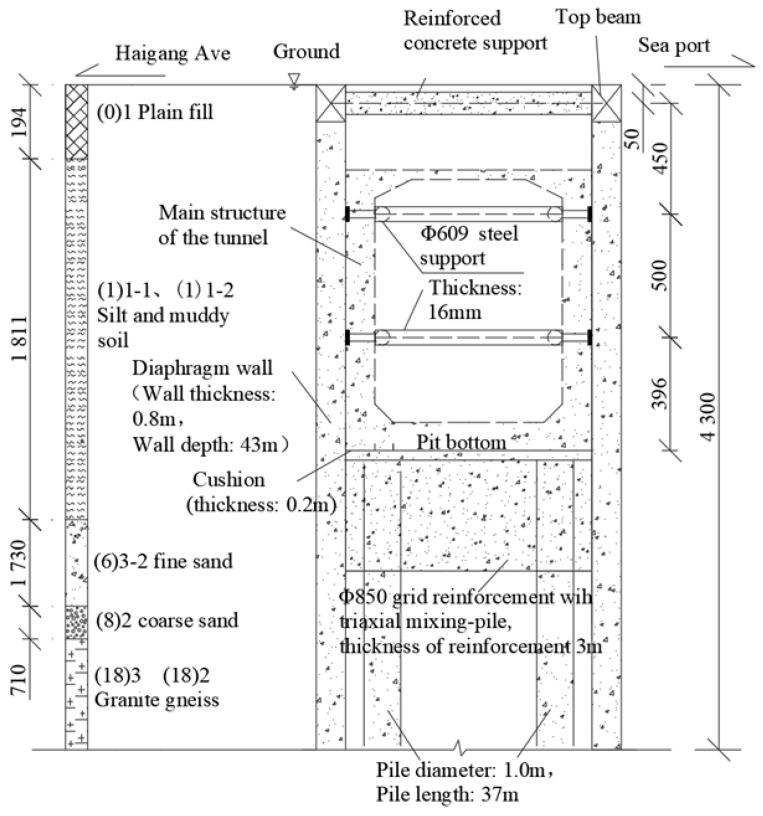
Profile of supporting structure system.

A typical K4 + 500 test section was selected, which uses a 0.8 m thick underground continuous wall and the first reinforced concrete support (800 mm × 800 mm) + two lanes Φ. The retaining structure system is composed of 609 steel supports (as shown in Figure 2) and has a wall depth of 43 m. The depth of the excavation is 13.96 m, the width of the excavation is 7.3 m, the groundwater depth is 1.9 m, and the depth of the ground elevation is 4.2 m. In order to ensure the stability of the main structure of the tunnel and the excavation of the foundation pit, soil reinforcement should be carried out at the bottom of the pit before excavation: (1) The bottom of the excavation is reinforced with Φ850 @ 600 triaxial mixing piles, and the construction process uses the “two mixing and two spraying” method. The cement content is 20%, the reinforcement width is 3.7 m, the spacing is 3.7 m, and the reinforcement depth is within 3 m below the pit bottom. (2) Bored cast-in-place bearing piles (also serve as anti-floating piles) are installed in the pit, with a designed pile diameter of 1.0 m and a pile length of 37 m. The longitudinal spacing of the tunnel route varies from 3 to 8 m, and two piles are arranged in the transverse direction with a spacing of 5.2 m, arranged in a rectangular shape.

### 2.3. Description and Cause Analysis of Water Gushing at the Bottom of the Pit

**Figure 3:**
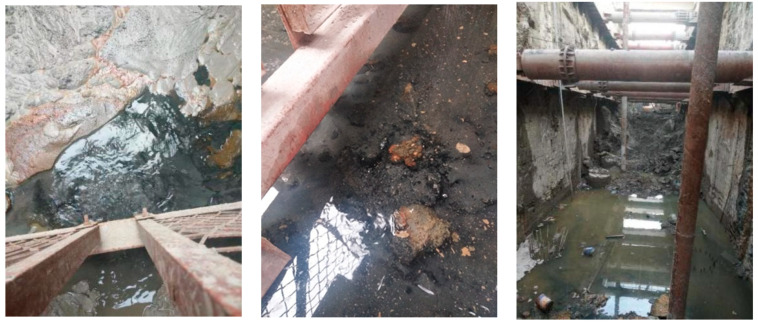
Actual scene of water gushing in the pit bottom.

On 6 December 2019, the foundation pit near the K4 + 500 mileage was excavated to the design elevation. To ensure the stability of the foundation pit and the safety of the main structure of the tunnel construction, the overall anti-floating stability of the pit bottom was checked before pouring the base cushion. Then, the deep pipe of the decompression well was backfilled with sand and sealed with concrete, and finally, the pipe was pulled out using a crane. At this point, there was water seepage, mud rolling, sand flow, and soil flow phenomena at the export of the pressure-reducing well. The gushing of groundwater contained silted soil and fine sand, and the water quality was turbid. After a few hours, it began to become clear. The mud around the pipe mouth floated, fine sand particles deposited, and the water accumulation in the pit was severe. After several consecutive days, the water gushing gradually decreased. All these caused construction stagnation on site and seriously affected the construction progress. The on-site photos are shown in Figure 3.

The geological survey data show that the main rock and soil layers within the excavation depth of the foundation pit at this section and the influence range of the underlying layer are plain fill, silt (mucky soil), fine sand, coarse sand, and granite gneiss, as shown in Figure 1, where the fine sand and coarse sand contain confined water locally. It was preliminarily speculated that the main reason for the water gushing at the bottom of the pit this time was due to the excavation process of the foundation pit. After the precipitation stabilized, the water head loss inside and outside the pit formed a water level difference, which was affected by local confined water. The groundwater outside the pit seeped into the pit by bypassing the wall foot or wall joint, generating a certain amount of uplift and seepage force upward along the pit bottom. In addition, after the removal of the pressure-reducing well casing, the backfill concrete and the filling materials such as silt and fine sand particles inside the pipeline, due to the influence of groundwater, resulted in poor cement bonding effect, causing local defects or weak areas at the bottom of the pit. Such an area is prone to stress concentration, creating favorable conditions for the formation of potential water-gushing channels. Under the combined action of pressured water and permeability force, loose fine particles and silt were suspended and washed away, migrating to the weakest areas. The gaps in the pipeline gradually increased and formed a connection, causing local damage to the soil structure and the loss of water resistance. The confined water in the weak permeable layer penetrated the holes and caused seepage damage, leading to the formation of small-scale permeable water gushing at the bottom of the pit.

## 3. Stability Analysis of Anti-Surge in Foundation Pits

### 3.1. Establish a Foundation Pit Surge Model

**Figure 4:**
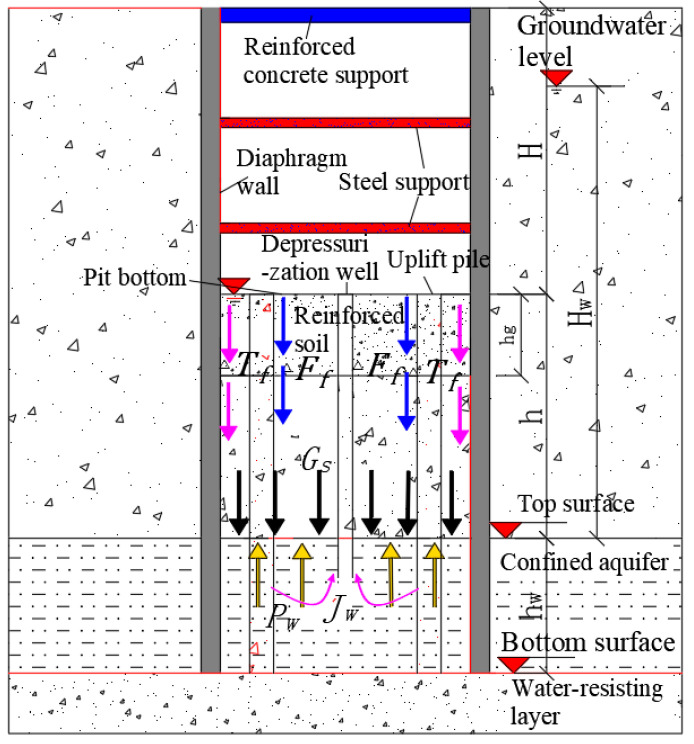
Analysis model of gushing in a foundation pit.

According to the case analysis of water gushing in the foundation pit, the seepage damage caused by local defects in the bottom plate, which leads to water leakage at the bottom of the pit, needs to be taken seriously. To this end, by establishing a seepage failure model for foundation pits, the safety factor for anti-surge and the calculation formula for the critical thickness of foundation pits can be derived. The analysis model of the foundation pit surge is shown in Figure 4. As shown in Figure 4, the excavation depth of the foundation pit is *H*, the self-weight of the impermeable layer soil is *G*_s_, the jacking force of the confined water is *P_w_*, the permeability force is *J*_w_, the shear resistance of the soil around the pit is *T*_f_, and the pile–soil friction resistance is *F_f_*.

The model is based on the following assumptions: (1) In the small deformation range, the waterproof layer of the baseboard at the bottom of the foundation pit is considered a homogeneous elastic body, and the reinforced soil in the passive zone is considered a homogeneous and continuous body. (2) For narrow and long strip foundation pits, the plane size effect of the foundation pit is not considered, which satisfies the plane section assumption. (3) The influences of excavations on soil disturbance and groundwater on the shear strength of soil are not considered.

### 3.2. Evaluation Method for Anti-Surge of Foundation Pits Considering Multiple Factors

#### 3.2.1. The Self-Weight of the Soil in the Impermeable Layer

In areas with deep soft soil, the extensive layers of silt and muddy soil present in the formation suggest that the soil characteristics within the foundation pit excavation range are relatively uniform. By considering the soil reinforcement within the pit and taking the bottom of the pit as the reference plane, the self-weight of the soil in the waterproof layer at the bottom of the pit can be determined.

(1) $$G_{s}=\int_{0}^{h_{g}}\gamma_{s}^{+}BLdh+\int_{h_{g}}^{h}\gamma_{s}BLdh=BL[\gamma_{s}^{+}h_{g}+\gamma_{s}(h-h_{g})]\;$$

where *γ*_S_ is the weight of the soil before the reinforcement of the waterproof layer (kN/m^3^), *γ*_S_^+^ is the weight of the soil after the reinforcement of the waterproof layer, *B* represents the excavation width of the foundation pit (m), *L* represents the length of the foundation pit (m), and the unit length *L* = 1.0 m is taken. h_g_ represents the thickness of the soil reinforcement (m) and *h* is the thickness of the waterproof layer (m).

#### 3.2.2. Upward Lifting Force of Confined Water at the Bottom of the Pit

During the dewatering and excavation process of the foundation pit, a head difference is formed between the inside and outside of the pit, and the head pressure of the confined water is generated, resulting in an upward lifting force at the bottom of the pit, namely:

(2) $$P_{w}=\gamma_{w}BLh_{w}$$

where *γ_w_* is the weight of the water (kN/m^3^), *h_w_* is the depth of the confined water (m), and the other parameters are the same as above.

#### 3.2.3. Permeability of Soil at the Bottom of the Pit

For the water pressure in the passive zone of the pit, the permeability has a lifting effect on the soil mass; hence, the soil permeability within the thickness range of the impermeable layer is:

(3) $$J_{w}=\gamma_{w}iBLh=\gamma_{w}BL\left(\frac{H_{w}-h}{H_{w}+2h_{w}+h}\right)h$$

where *H*_w_ is the elevation of the groundwater level (m), and the other parameters are the same as Equation (1).

#### 3.2.4. Shear Resistance of Soil around the Pit

In the soft soil layer, considering the beneficial effect of soil shear strength on the resistance to sudden surges, according to the Mohr–Coulomb theory, the shear strength at any point along the shear plane at the depth hg of the reinforced soil is:

(4) $$\tau_{f}^{+}=\sigma_{n}\tan\varphi^{+}+c^{+}=K_{0}\gamma_{s}^{+}h_{g}\tan\varphi^{+}+c^{+}$$

where *φ* and *φ*^+^ are the internal friction angle of the soil before and after reinforcement (°) and *c* and *c*^+^, respectively, represent the cohesive force of the soil before and after reinforcement (KPa).

Therefore, the shear resistance of the soil along the reinforcement depth range *h_g_* is:

(5) $$\Delta T_{f}^{+}=2(B+L)\int_{0}^{h_{g}}\tau_{f}^{+}dh=2(B+L)[\frac{K_{0}\gamma_{s}^{+}}{2}h_{g}{}^{2}\tan\varphi^{+}+c^{+}h_{g}]$$

Similarly, the shear resistance of the soil within the range of the depth (*h*–*h_g_*) of the unreinforced soil layer is obtained:

(6) $$\Delta T_{f}=2(B+L)\int_{h_{g}}^{h}\tau_{f}dh=2(B+L)[\frac{K_{0}\gamma_{s}}{2}{\left(h-h_{g}\right)}^{2}\tan\varphi +c(h-h_{g})]$$

The total lateral friction force of the soil within the thickness range of the impermeable layer is:

(7) $$T_{f}=\Delta T_{f}^{+}+\Delta T_{f}$$

#### 3.2.5. Frictional Resistance between Piles and Soil

During the excavation process of the foundation pit, there is a certain shear frictional resistance between the pile side and the reinforced soil due to the influence of the uplifting force of confined water and seepage force caused by the water head. In practical engineering [3], the frictional resistance between piles and soil is generally calculated based on the contact area of a single pile and reinforced soil at the maximum pile spacing. Namely:

(8) $$F_{f}=uh_{g}f_{sp}=\pi dh_{g}f_{sp}$$

where *u* is the perimeter of the pile foundation section (m); *u* = *πd*, *d* is the pile diameter (m); *f*_sp_ is the frictional resistance per unit area (kN), which is usually taken as 40 kN/m^2^ for cast-in-place piles; and *h_g_* is the depth of the reinforced soil.

### 3.3. Critical Thickness of Anti-Surge

Previous studies have shown [15] that when considering the parameters of reinforced soil, only the increase in cohesion of the shear strength of the reinforced soil needs to be considered and the changes in soil weight and the internal friction angle before and after reinforcement can be ignored, namely:

(9) $$\gamma_{s}^{+}=\gamma_{s},\;\varphi^{+}=\varphi$$

According to the vertical limit equilibrium conditions of the impermeable layer, the safety factor *K_S_* for anti-surge is obtained:

(10) $$K_{s}=\frac{G_{s}+T_{f}+F_{f}}{P_{w}+J_{w}}$$

Then:

(11) $$K_{s}=\frac{BL\gamma_{s}h+BLK_{0}\gamma_{s}\tan\varphi \left[h_{g}^{2}+{\left(h-h_{g}\right)}^{2}\right]+2\left(B+L\right)\left[h_{g}\left(c^{+}-c\right)+hc\right]+\pi dh_{g}f_{sp}}{\gamma_{w}BL\left[\left(\frac{H_{w}-h}{H_{w}+2h_{w}+h}\right)h+h_{w}\right]}$$

Numerous studies have shown that the influence of the internal friction angle on the shear strength of soil in soft soil areas can be ignored. Therefore, the above equation is further rewritten as:

(12) $$K_{s}=\frac{2(B+L)[h_{g}(c^{+}-c)+hc]+\mathrm{BL}y_{s}h+\pi dh_{g}f_{sp}}{\gamma_{w}BL[[\frac{H_{w}-h}{H_{w}+2h_{w}+h}]h+h_{w}]}=\frac{\gamma_{s}}{\gamma_{w}\left(\frac{H_{w}-h}{H_{w}+2h_{w}+h}+\frac{h_{w}}{h}\right)}+\frac{2\left(B+L\right)\left[h_{g}\left(c^{+}-c\right)+hc\right]+\pi dh_{g}f_{sp}}{\gamma_{w}BL\left[\left(\frac{H_{w}-h}{H_{w}+2h_{w}+h}\right)h+h_{w}\right]}$$

The above equation is then simplified to obtain:

(13) $$\alpha \cdot h^{2}+\beta \cdot h+\eta =0$$

where $\alpha =-2c(B+L)-BL(\gamma_{w}K_{s}+\gamma_{s})$,

$$\begin{array}{l} \beta =BL[\gamma_{w}K_{s}(H_{w}+h_{w})-\gamma_{s}(H_{w}+2h_{w})]-2(B+L)[h_{g}(c^{+}-c)+c(H_{w}+2h_{w})]-\pi dh_{g}f_{sp}\\ \eta =\left(H_{w}+2h_{w}\right)\left[\gamma_{w}h_{w}BLK_{s}-2h_{g}\left(B+L\right)\left(c^{+}-c\right)-\pi dh_{g}f_{sp}\right] \end{array}$$

The critical thickness *h_cr_* that satisfies the anti-surge safety factor *K_s_* is:

(14) $$h_{cr}=\frac{-\beta \pm \sqrt{\beta^{2}-4\alpha \eta }}{2\alpha }$$

### 3.4. Model Validation

Based on the K4 + 500 test section, the excavation width of the foundation pit *B* is =7.3 m, and the average soil cohesive force of the strata within the excavation depth of the foundation pit is *c* = 9.2 kPa. According to the research conclusion of Lv [16], the cohesive force *c*^+^ of cemented soil in the passive zone is taken as 15 kPa, and the other parameter values are the same as above. The minimum safety factor *K*_s_ for anti-surge calculated using Formula (12) is 1.455. This value is slightly higher than 1.2*γ*_0_, which is the allowable minimum requirement in the “Technical Specification for Building Foundation Pit Support Engineering in Guangdong Province” (DBJ/T15-20-97). *γ*_0_ is the importance coefficient of the foundation pit. The safety level of the foundation pit in this project is Level 1 and *γ*_0_ = 1.1; thus, as calculated using Formula (14), the critical thickness of anti-surge *h*_cr_ = 5.87 m. It can be seen that the theoretical derivation results of this article have a certain degree of rationality.

## 4. Parameters Influences Analysis

### 4.1. Soil Shear Strength

**Figure 5:**
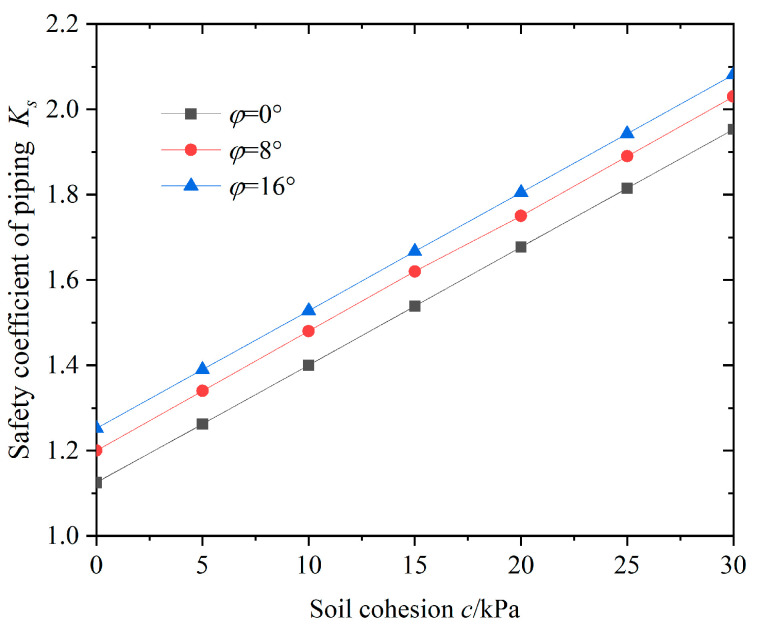
Relationships between *c* and *K_s_*.

The shear strength of the impermeable layer of soil at the bottom of the foundation pit plays an important role in the stability of resisting surge. In practical engineering, using deep cement mixing or grouting to reinforce the soil inside the pit is an effective measure to deal with surges caused by confined water [13,14]. The variation in the anti-surge safety coefficient at the bottom of the pit under different soil cohesions is shown in Figure 5.

As shown in Figure 5, the safety factor against sudden surge increases linearly with the increase in soil cohesion in the impermeable layer, and the stability of the foundation pit against sudden surge is significantly improved. When the cohesion of the soil increases from 0 to 30 kPa, the safety factor Ks against sudden surge increases by 69.2%. At the same time, under the same cohesive force conditions, the anti-surge safety coefficient slowly increases with the increase in the internal friction angle of the soil, and the growth rate slightly decreases. This indicates that the cohesive force of the soil has a greater impact on the anti-surge stability compared with the internal friction angle. It can be seen that using reinforcement measures at the bottom of the pit to improve the shear strength characteristics of the soil can effectively improve the stability of the foundation pit against sudden surges. This can also reduce the uplift deformation at the bottom of the pit and the horizontal displacement of the retaining structure, thus achieving effective control of the excavation pit bottom to prevent sudden surges, and deformation of the retaining structure, the surrounding rock, and the soil environment.

**Figure 6:**
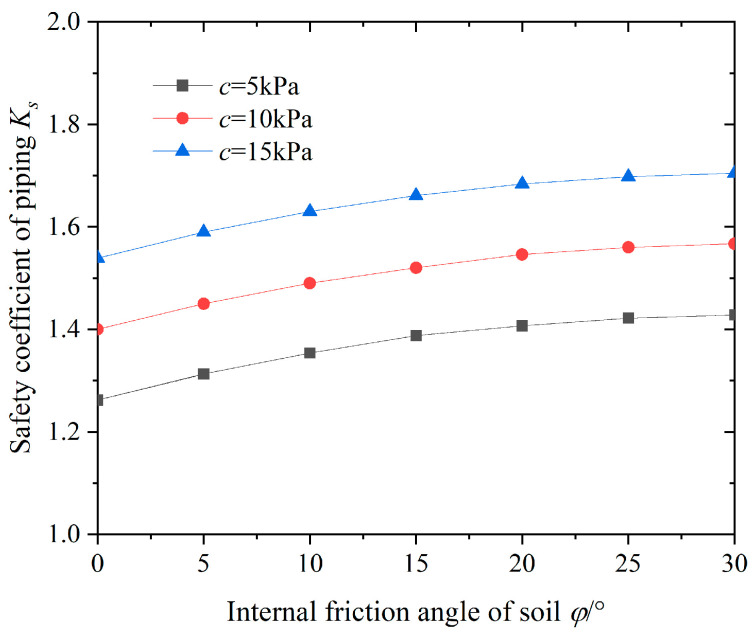
Relationships between *φ* and *K_s_*.

Next, we analyze the impact of the internal friction angle of the impermeable soil layer *φ* on surge safety factor *K*s. The variation pattern in the anti-surge safety coefficient at different internal friction angles is shown in Figure 6.

From Figure 6, it can be seen that as the internal friction angle of the impermeable soil layer *φ* increases, the surge resistance safety coefficient *K*_s_ increases. It shows a non-linear growth trend, mainly manifested by a uniform growth rate in the early stage and gradually stabilizing in the later stage. When the internal friction angle *φ* > 20°, the change in safety factor is not significant. In addition, when *φ* is invariant, the anti-surge safety coefficient increases uniformly with the increase in soil cohesion. During the process of increasing the internal friction angle of the soil from 0° to 30°, the anti-surge safety coefficient *K*_s_ only increases by 11.9%, and the improvement in the anti-surge safety coefficient of the foundation pit is not significant. It can be seen that compared with the cohesive force of soil *c*, the impact of the internal friction angle *φ* on anti-surge stability is not significant, and the calculated value of the internal friction angle in the soil is much smaller than the influence of cohesion. The research conclusion of Zhang et al. [15] confirms this viewpoint, which also explains that the strength index of soil reinforcement generally considers the influence of soil cohesion as the main factor.

### 4.2. Depth of Soil Reinforcement in the Foundation Pit

**Figure 7:**
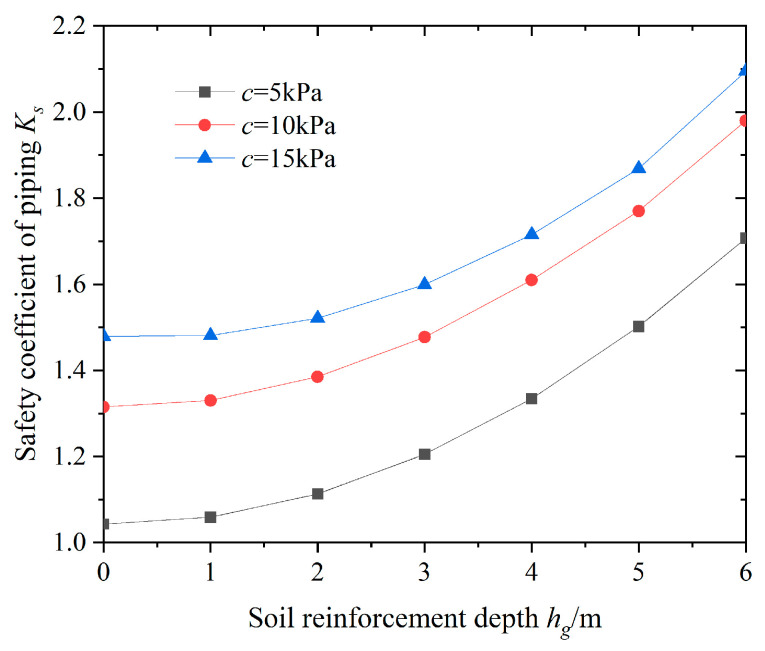
Relationships between *h_g_* and *K_s_*.

To study the effect of soil reinforcement depth in the pit on the stability of the bottom of the foundation against sudden surges, and under the principles of ensuring the safety and economy of the foundation pit, the variation in the anti-surge safety coefficient at the bottom of the pit under different soil reinforcement depth conditions was calculated, as shown in Figure 7.

From Figure 7, it can be seen that as the reinforcement depth *h_g_* of the soil in the pit increases, the anti-surge safety factor *K*_s_ shows a non-linear growth pattern, and the growth rate increases. When the reinforcement depth of the soil increases from 0 to 6 m, the anti-surge safety coefficient *K*_s_ increases by a maximum of 50.6%, and the anti-surge safety coefficient of the foundation pit is greatly improved. In addition, under the same soil reinforcement depth conditions, as the soil cohesion increases, the safety factor against surge increases, but the growth rate gradually decreases, indicating that the quality of the soil properties has a significant impact on the safety factor value. Obviously, improving the shear strength of the soil using reinforcement measures at the bottom of the pit is beneficial for improving the stability of the pit against sudden surges.

### 4.3. Thickness of the Impermeable Layer

**Figure 8:**
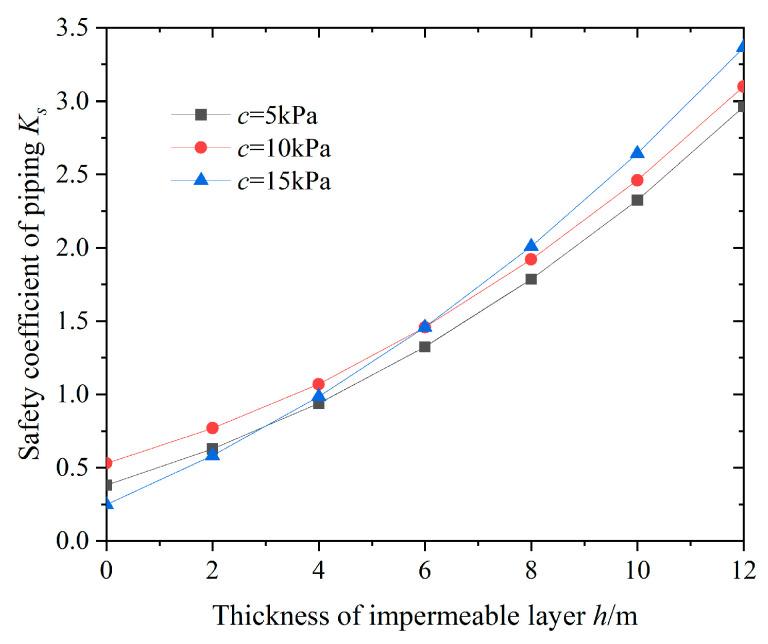
Relationships between *h* and *K_s_*.

To study the influence of the thickness of the impermeable layer on the stability of the bottom of the foundation pit against sudden surges, given different thicknesses of the impermeable layer, the variation in the safety factor against sudden surges at the bottom of the pit was calculated, as shown in Figure 8.

From Figure 8, it can be seen that the thickness *h* of the pit bottom waterproof layer and the anti-surge safety coefficient *Ks* exhibit a non-linear growth pattern. That is, the smaller the thickness of the pit bottom waterproof layer, the smaller the anti-surge safety coefficient. The larger the deformation of the pit bottom uplift, the worse the stability of the foundation pit, and the more likely there is a risk of surge. On the contrary, the thicker the waterproof layer at the bottom of the pit, the higher the safety factor against sudden surge, the weaker the impact of the confined water on the sudden surge stability, and the safer the excavation of the foundation pit, which is basically consistent with the research conclusions of Zhang et al. [17]. In addition, when the thickness of the impermeable layer increases from 0 to 12 m, the anti-surge safety factor *K_s_* increases by 484.9%, and the anti-surge safety factor of the foundation pit significantly improves. Therefore, for foundation pits containing confined water, increasing the thickness of the pit bottom waterproof layer, i.e., reducing the excavation depth of the foundation pit, is very beneficial for improving the stability of the pit bottom against sudden surges.

### 4.4. Diameter of the Bearing Pile in the Foundation Pit

**Figure 9:**
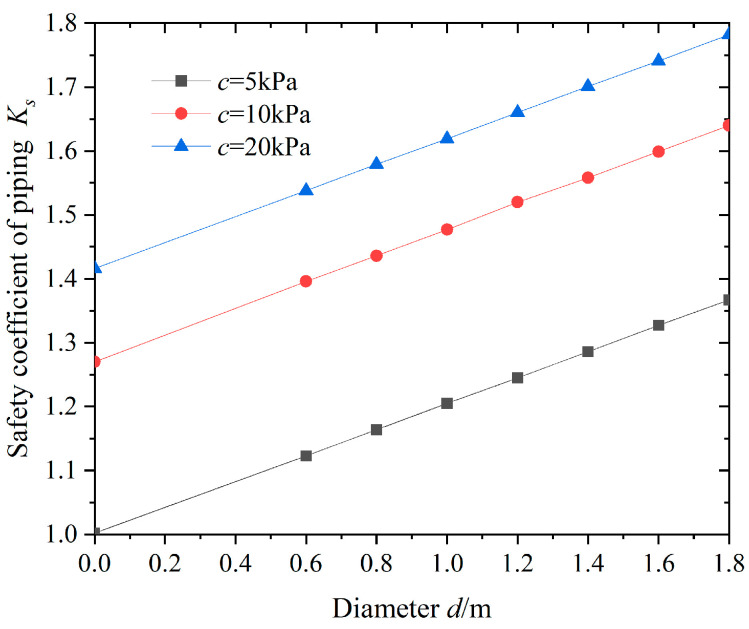
Relationships between *d* and *K_s_*.

To study the effect of the diameter of the bearing pile in the pit on the stability against surge, the variation law of the safety factor against surge at the bottom of the pit was calculated under different pile diameters, as shown in Figure 9.

From Figure 9, it can be seen that as the diameter *d* of the bearing pile in the pit gradually increases, the safety factor *K*s of anti-surge at the bottom of the pit shows a linear increasing trend. Under the same pile diameter conditions, as the soil cohesion increases, the anti-surge safety factor *K*s increases, but the growth rate gradually decreases. When the thickness of the impermeable layer increases from 0 to 1.8 m, the anti-surge safety factor *K_s_* increases by 29.1%, and the anti-surge safety factor in the foundation pit improves. The main reason is that the increase in pile diameter increases the contact area between the piles and soil, which is more conducive to the development of pile–soil frictional resistance and thus improves the stability of the foundation pit against sudden surges.

### 4.5. Height of the Confined Water Level

**Figure 10:**
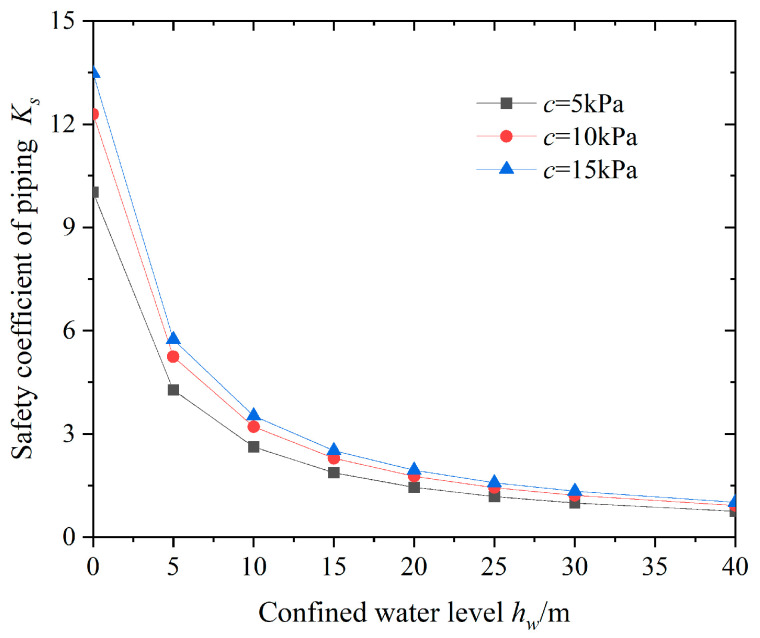
Relationships between *h_w_* and *K_s_*.

To study the effect of the height of the confined water level on the stability of sudden surges, the variation in the safety coefficient against sudden surges at the bottom of the pit was calculated under different confined water heads, as shown in Figure 10.

From Figure 10, it can be seen that as the height of the confined water level *h*_w_ increases, the anti-surge safety coefficient *K*_s_ at the bottom of the pit shows a non-linear decreasing trend, manifested as an accelerated decrease in the early stage and gradually tending to stabilize. The higher the height of the confined water level, the greater the pressure of the confined water, and the smaller the safety factor against sudden surges. That is to say, the poorer the stability against sudden surges, the greater the possibility of sudden surges, which is consistent with the viewpoint proposed by Li et al. [12]. In addition, at the same height of the confined water level, as the cohesion of the soil increases, the safety factor against sudden surge decreases, but the growth rate gradually decreases. Therefore, in the process of excavation and dewatering of the foundation pit, a reasonable reduction in the confined water level has a significant impact on the stability of the foundation pit.

## 5. Evaluation of the Treatment for Water Gushing at the Bottom of the Pit

### 5.1. Evaluation of the Treatment for Water Gushing at the Bottom of the Pit

**Figure 11:**
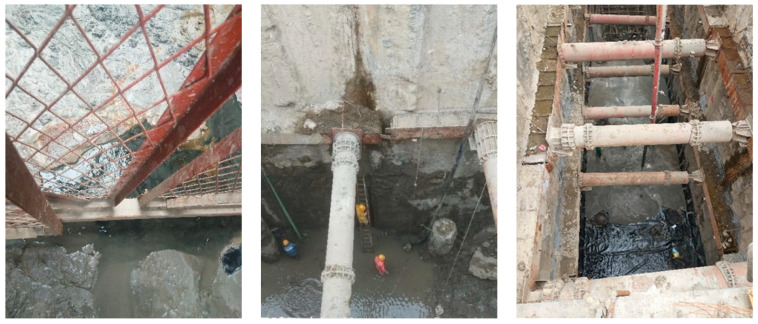
Pit bottom treatment and cushion pouring.

The characteristics and causes of the water gushing were analyzed, and the on-site treatment plan was used, which included backpressure at the bottom of the pit (manual ladder climbing), pumping water from the pit to the outside, and the timely sealing of the pit bottom cushion layer (laying waterproof rolls). Photos taken on site are shown in Figure 11. Considering that the stratum near the water gushing point contains confined water of the weak permeable layer, uneven settlement or local uplift deformation easily occurs when pouring the bottom plate, and the deformation monitoring conditions of the pit bottom were limited. In order to ensure the site treatment effect and the subsequent construction safety of the main structure, a new monitoring method representing the uplift deformation of the pit bottom is proposed: use the vertical pressure monitoring results at the bottom plate of the foundation pit to reflect the deformation behavior of the pit bottom and evaluate the uplift deformation.

### 5.2. Soil Pressure Test at the Bottom of the Foundation Pit

**Figure 12:**
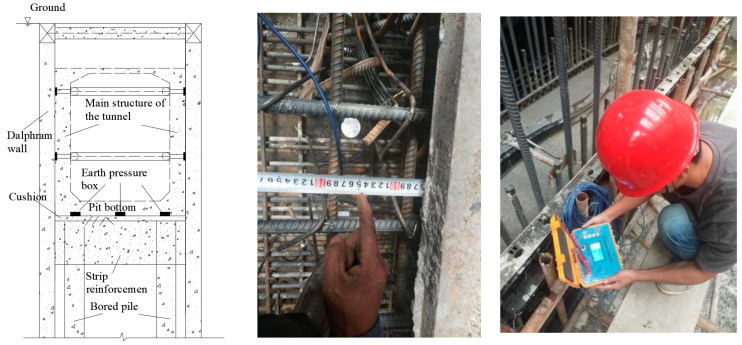
Earth pressure box layout and field data acquisition.

According to the on-site construction situation, after the concrete pouring of the bottom slab cushion was completed, a steel tape measure was used to determine the burial position of the soil pressure box. Using the embedding method, three soil pressure sensors (model BK-1260S with a testing accuracy of 0.001 MPa) were installed on the surface of the flat cushion located in the middle of the pit bottom (measuring point number 07631) and about 1.0 m away from the side wall on both sides (measuring point number 44399 near the Haigang Avenue and 12,400 near the Haigang Wharf). At the same time, an appropriate amount of fine sand was backfilled around the soil pressure box, and the testing element cables were bundled along the main reinforcement of the bottom plate and led out along the main reinforcement of the side wall. The end joints of the wires were protected to prevent compression damage, blockage, or water ingress. During the concrete pouring process of the main structure of the tunnel, real-time data were manually collected using an intelligent reading instrument. The arrangement of the earth pressure box and work photos taken on site are shown in Figure 12.

### 5.3. Analysis of Monitoring Data

**Figure 13:**
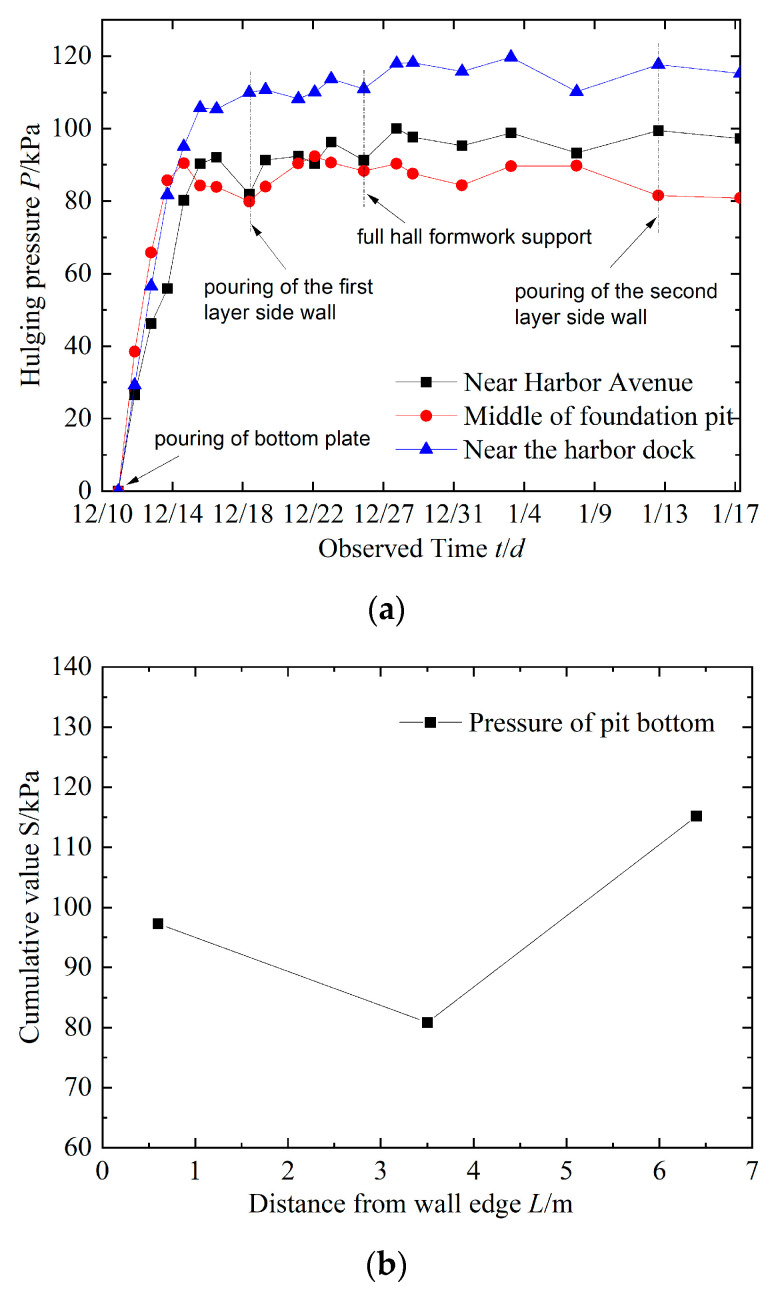
Pit bottom pressure monitoring results. (**a**) Time history curve of the pit bottom pressure. (**b**) Cumulative change curve of the pit bottom pressure.

According to the on-site construction progress, the data collected on site were analyzed and processed, and the monitoring results are shown in Figure 13.

Figure 13 shows that the changes in pressure at the bottom of the pit at each measuring point are basically consistent. In the early stage, it accelerates and reaches its peak after about a week, followed by a fluctuating trend with a small amplitude of change, indicating that the pit bottom is in a stable state with small uplift and deformation, and the on-site treatment effect is good. At the same time, the maximum pressure at the bottom of the pit is 115.189 kPa (near the harbor dock), and the change in the measurement point in the middle of the pit is the smallest with a minimum value of 80.834 kPa. The difference between the two is about 30%, which manifests as large at both ends and small in the middle, showing a “V” shaped stress pattern. If the bottom plate is considered a “soil beam” structure, it is considered that the stress and uplift deformation of the bottom plate develop in harmony. Obviously, compared with a conventional foundation pit, the narrow and long foundation pit in this project has a different “inverted pot” shape of raised deformation at the bottom. The main reasons are as follows: (1) Compared with a wide foundation pit, the narrow and long foundation pit in this project has stronger resistance to lateral deformation and better overall stability. The research conclusions of LI et al. [18] and Zhuang et al. [19] all confirm this viewpoint. (2) Under the external soil pressure of the foundation pit wall, the connecting wall compresses toward the pit, causing a vertical uplift of the soil at the bottom of the pit. However, due to the reinforcement of the passive soil in the pit and the “active restraint” effect of the anti-uplift pile, the overall stiffness of the pit bottom soil and the surrounding system is improved, and the rebound at the bottom of the pit is effectively controlled. In addition, with the influence of the self-weight of the main structures on both sides, the force in the middle of the foundation pit is smaller than that on both sides, which is conducive to the stability of the pit bottom against uplift.

### 5.4. Calculation of Vertical Deformation of the Pit Bottom Uplift

According to the research conducted by Wang et al. [20], it is believed that there is no significant spatial effect on the deformation of narrow and long foundation pits, which satisfies the plane section assumption. Therefore, considering the influence of lateral pressure on the soil at the bottom of the pit, the formula for calculating the vertical strain of the soil at the bottom of the pit is:

(15) $$\begin{array}{ll} \varepsilon_{z} & =\frac{1-\mu^{2}}{E_{p}}\left(\sigma_{z}-\frac{\mu }{1-\mu }\sigma_{x}\right)\\ & =\frac{1-\mu^{2}}{E_{p}}\left(\sigma_{z}-\frac{\mu }{1-\mu }K_{0}\gamma D\right) \end{array}$$

where *γ* is the soil weight (kN/m^3^), *D* is the excavation depth of the foundation pit (m), *μ* is Poisson’s ratio, *K*_0_ is the coefficient of the lateral earth pressure of soil, where K_0_ = 1− sin*φ*, *φ* is the internal friction angle of the soil (°), *E*_p_ is the rebound modulus of the bottom slab (MPa), and *σ*_z_ is the vertical stress at the bottom of the pit (MPa).

Considering the bottom plate of the foundation pit as a homogeneous elastic body, according to the theory of material mechanics:

(16) $$\varepsilon_{z}=\frac{\Delta \delta }{\delta }$$

where Δδ is the vertical deformation of the bottom plate (mm) and δ is the thickness of the bottom plate (mm) where, according to the design scheme, δ=700 mm.

**Figure 14:**
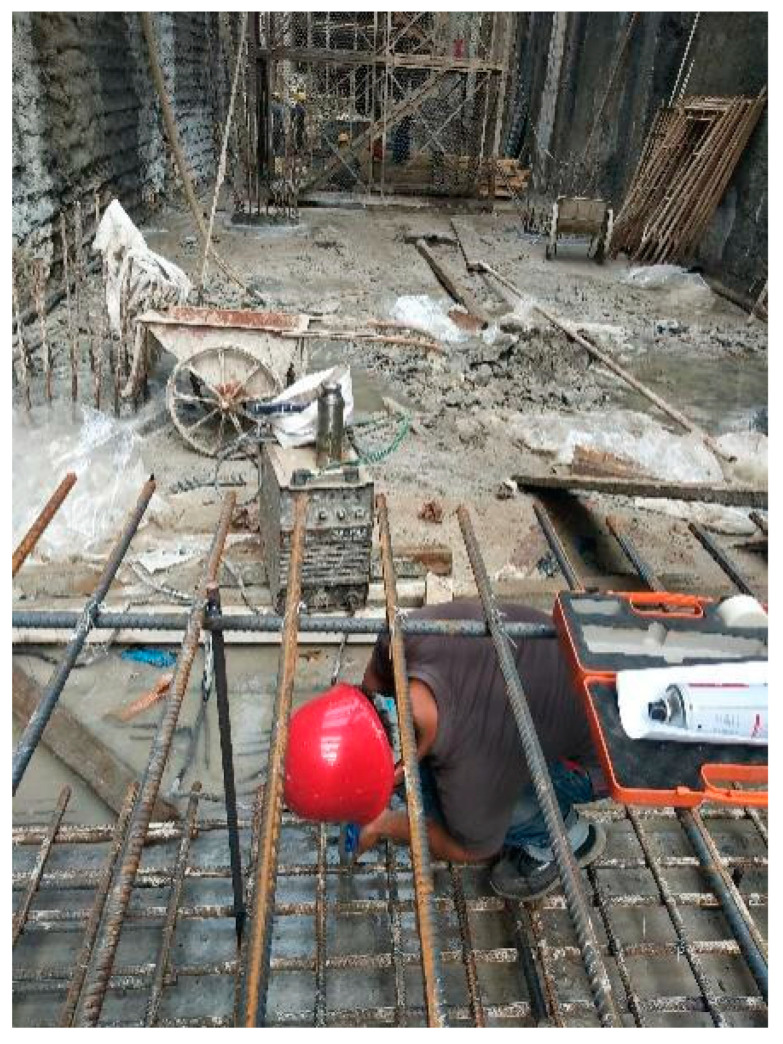
Field rebound test.

Considering that the test section has a thick layer of silt and muddy soft clay, it is believed that there is little difference in soil properties within the excavation range of the foundation pit and *γ* = 17.5 kN/m^3^, *μ* = 0.35, *φ* = 8°, and the depth of the foundation pit *D* = 13.96 m. The vertical uplift pressure at the bottom of the pit was obtained with on-site testing, and the deformation modulus of the cushion layer in the pit was obtained with rebound testing using a concrete rebound instrument. The test results are shown in Table 2, and the on-site rebound test photos are shown in Figure 13. The field rebound test is shown in Figure 14.

According to Formula (15), the vertical rebound strain of the soil at the bottom of the pit is $\varepsilon_{z}=2.009\times 10^{-3}$. Substituting this value into Formula (16), the deformation of the foundation pit bottom plate was calculated, Δδ=1.406mm, and the deformation is very small. The pit bottom is in a stable state, indicating that the on-site treatment effect is good.

## 6. Conclusions

(1) For this project case, the minimum safety factor against surge considering multiple factors is 1.455, and the critical thickness is 5.87 m, which is in line with the specification.

(2) The height of the confined water level, the thickness of the impermeable layer, the shear strength of the soil, the depth of soil reinforcement in the pit, and the diameter of the bearing pile have varying degrees of influence on the safety factor of the foundation pit against surge. In practical engineering, measures such as soil reinforcement in the passive area of the pit, increasing the thickness of the impermeable layer, and reducing the bearing water level can effectively improve the stability of the foundation pit surge resistance.

(3) In this paper, a new monitoring method to characterize the deformation of pit bottom uplift is presented. The pit was obtained with the pit bottom counter-pressure, pumping water out of the pit, and the timely construction of the pit bottom bedding to block the program. The measured maximum pressure of the bottom plate is 115.189 kPa, and the deformation corresponding to the method proposed in this paper is 1.406 mm, which is better disposed in the field.

## Figures and Tables

**Table 1:** Physical–mechanical parameters of soil layer.

Stratum Name	*d*/m	*γ*/(kN·m^−3^)	*c*/kPa	*φ*/°	*E*/MPa
plain fill	3.9	18.5	10.2	7.0	3.5
silt	21.1	17.1	7.8	7.5	2.1
mucky soil	10.3	17.5	10.6	9.0	2.4
muddy silt	5.8	18.1	0	15.5	3.8
silty clay	14.0	18.7	16.0	18.0	4.8
silt	6.4	19.0	0	25.6	12
fine sand	12.2	19.0	0	27.2	16.5
middle sand	3.5	19.5	0	28	21
coarse sand	4.8	20.0	0	29.5	24
gravel	5.2	21.0	0	32	30
fine gravel	2.9	21.0	0	34	32
granite gneiss	18.0	24.0	120	39	400

**Table 2:** Field rebound test record.

Test Area	Rebound Value/MPa				Mean Value/MPa
**K4 + 500**	**14.2**	**18.1**	13.5	13.3	14.6
	14.4	16.4	18.2	14.5	
	12.7	16.3	14.0	12.3	
	15.0	16.2	14.2	12.4	

## Data Availability

Some or all data, models, or code generated or used during this study are available from the corresponding author upon request.
